# Inhibition of primary cilia-hedgehog signaling axis triggers autophagic cell death and suppresses malignant progression of VHL wild-type ccRCC

**DOI:** 10.1038/s41419-024-07085-8

**Published:** 2024-10-10

**Authors:** Shuo Tian, Songliang Du, Chenfeng Wang, Yu Zhang, Hanfeng Wang, Yang Fan, Yu Gao, Liangyou Gu, Qingbo Huang, Baojun Wang, Xin Ma, Xu Zhang, Yan Huang

**Affiliations:** 1 Department of Urology, The Third Medical Center, Chinese PLA General Hospital, Beijing, 100853 China; 2 Chinese PLA Medical School, Beijing, China

**Keywords:** Renal cell carcinoma, Cancer prevention, Cancer immunotherapy

## Abstract

Primary cilia are present on renal tubules and are implicated to play a pivotal role in transducing signals during development; however, the oncogenic role of cilia in clear cell renal cell carcinoma (ccRCC) has not been examined. Here we show that VHL wild-type ccRCC cell lines have a high incidence of primary cilia, and a high frequency of primary cilia is positively correlated with VHL expression and poor prognosis. Besides, the depletion of KIF3A and IFT88, genes required for ciliogenesis, significantly inhibited tumor proliferation and metastasis in vitro and in vivo. Further analysis found that mutations of key genes in hedgehog signaling are enriched in VHL wild ccRCC, its downstream signaling activation depends on ciliogenesis. Moreover, depletion of primary cilia or suppression of hedgehog pathway activation with inhibitor-induced robust autophagic cell death. Collectively, our findings revealed that primary cilia could serve as a diagnostic tool and provide new insights into the mechanism of VHL wild-type ccRCC progression. Targeting the primary cilia-hedgehog pathway may represent an effective therapeutic strategy for VHL wild-type ccRCC.

## Introduction

Renal cell carcinoma (RCC) accounts for 90% of adult renal malignancies and is the most lethal urologic malignancy with increasing incidence worldwide [1]. Clear cell RCC (ccRCC) is the most common and aggressive type of renal malignancy, accounting for 70% to 80% of RCC cases. Inactivation of the von Hippel Lindau (VHL) gene is a hallmark of the majority of cases of sporadic ccRCC, and the change in its downstream HIF-VEGF signaling pathway results in the use of antiangiogenic targeted therapies, which are now first line in the treatment of advanced renal tumors. However, VHL wild-type (VHL-wt) ccRCC at advanced stages has a poor prognosis because of distal metastasis and less targeted-therapy effectiveness, emphasizing the urgent need to clarify the underlying mechanisms and find better therapeutic targets.

Primary cilia are microtubule-based organelles that behave like a cellular antennae controlling key signaling pathways during development and tissue homeostasis. Since primary cilia require the same structural components as chromosome segregation, they are usually considered to act as a brake on cell proliferation, migration and invasion [2, 3]. Thus, loss of primary cilia has been associated with onset and progression of several human tumors [4–6], providing an advantage for these cancer cells to freely proliferate [4]. In contrast, basal cell carcinomas and medulloblastoma depend on retention of cilia to activate Hedgehog (Hh) signaling and promote tumorigenesis when tumor-inducing event is oncogenic mutation of Smo. Besides, enhanced ciliogenesis in cancer cells was found to increase Hh signaling and facilitate resistance to a number of kinase inhibitors [7–9]. In addition, pVHL has been shown to localize to the ciliary axoneme [10–12], and knockdown of pVHL and GSK3beta would impede the formation of primary cilia. In a mouse model of ccRCC induced by combined deletion of VHL, Trp53 and Rb1, the frequency of ciliation in tumor cells is significantly reduced [13], and loss of primary cilia was associated with onset of premalignant cysts and renal cancer [14–16], providing compelling evidence that the primary cilium functions as a tumor suppressor organelle in the kidney. However, the incidence and role of primary cilia in VHL wild-type ccRCC remains unelucidated.

Functioning of the primary cilium has been recently connected to autophagy, a highly conserved intracellular process for degradation of cellular components in lysosomes. Many studies advocated that the primary cilium is required for autophagy activation [17, 18] while some cancer cells such as pancreatic ductal adenocarcinoma and thyroid Hürthle cell carcinoma (XTC.UC1) showed increased autophagy activity despite absence of primary cilia [19]. Whereas, autophagy could degrade proteins that contribute to ciliary growth as well as regulatory proteins that block ciliogenesis [20]. The interplay between primary cilia and autophagy remains complicated and depends on cell types or cell conditions. Mounting evidence demonstrated that Hh signaling from the primary cilium also plays a dual role in regulating autophagy [20]. Some studies showed that Hh signaling inhibits autophagy and Hh inhibition induces autophagy and promotes autophagic cell death [21], while other research indicated that Hh signaling activation upregulates autophagy to promote progression and drug resistance in breast and pancreatic cancer [22–24]. Therefore, the crosstalk between primary cilium-Hh signaling axis and functional autophagy in cancer progression remains unclear and identification of underlying mechanism may provide new therapeutic targets with clinical applicability.

In this study, we report that primary cilia are preserved in VHL-wt ccRCC and play an oncogenic role in the progression of VHL-wt ccRCC. High incidence of ciliation correlates with higher risk of distal metastasis and depletion of ciliary genes significantly impairs proliferation and metastasis of VHL-wt ccRCC. Further studies show that mutations of key genes in hedgehog pathways are enriched in VHL-wt ccRCC, its downstream signaling activation depends on ciliogenesis. Mechanistic dissection reveals that loss of primary cilia or inhibition of hedgehog signaling induces robust autophagic cell death, providing promising therapeutic targets for treatment of VHL-wt ccRCC.

## Materials and methods

### Human samples

215 cases of human ccRCC tissues were obtained from the clinical database of Chinese PLA General Hospital. Informed consent was obtained from each patient and the study was approved by the Institutional Review Board of Chinese PLA General Hospital. All samples of cancer tissue had been pathologically confirmed as ccRCC according to the 2011 Union for International Cancer Control TNM classification of malignant tumors. All patients were informed and signed a consent on the use of clinical specimens for scientific research. Tissue microarray was constructed in our laboratory.

### Cell culture and drug exposure

Human Embryonic Kidney Cell HEK293TN cell, human ccRCC cell lines SN12-PM6, Caki-1, Caki-2, 786-O, 769-P, A498, and normal kidney cell lines HK2, HKC cells were originally purchased from the National Platform of Experimental Cell Resources for Sci-Tech. SN12-PM6 cells were preserved in our laboratory. Caki-1, Caki-2 were cultured in McCoy 5 A medium (HyClone), 786-O, 769-P, A498, HK2, HKC were cultured in RPMI 1640 (Gibco) and SN12-PM6, HEK293TN cells were cultured in DMEM (HyClone) with 10% FBS (Gibco), respectively. All cells were cultured in a 5% CO_2_ incubator at 37 °C. The GLI1/2 inhibitor GANT61 and the SMO inhibitor Erismo were obtained from Selleck Chemicals (Houston, TX, USA). Stock solutions were prepared in dimethyl sulfoxide (DMSO) and stored at −20 °C. Working solutions were prepared immediately before use and control cells were treated with the corresponding drug solvent.

### Western blot and antibodies

Cells were lysed using radioimmunoprecipitation assay (RIPA; Beyotime, Shanghai, China) buffer. The total protein concentration was measured using a BCA Protein Assay Kit. Equal amounts of total protein were separated by SDS-PAGE and transferred to 0.25 μm PVDF membranes (Millipore, Billerica, USA). Primary antibodies included anti-β-actin (1:5000, 66009-1-Ig; Proteintech); anti-VHL (1:500, sc135657; Santa Cruz); anti-P62 (1:10000, ab109012, Abcam); anti-LC3B (1:2000, ab192890; Abcam), anti-KIF3A (1:1000, 13930-1-AP; Proteintech); anti-IFT88 (1:1000, 13967-1-AP; Proteintech); anti-GLI1 (1:2000, 66905-1-Ig; Proteintech). After washing thrice with TBST, the membranes were incubated with an HRP-conjugated (horseradish peroxidase conjugated; the Promoter Biotechnology, Wuhan, China) secondary antibody at a 1:5000 dilution for 1 h at room temperature. Antibody binding was detected using Super Signal West Pico Chemiluminescent Substrate (Thermo Scientific). Experiments were repeated at least 3 times, and band intensities were quantified using ImageJ software.

### MTT assay

The cells in different groups were seeded into 96-well plates (3000 cells per well). Absorbance was measured at 24, 48, 72, and 96 h after seeding at 37 °C with 5% CO_2_. The viability of cells was assessed using 20 μL MTT reagent (Cell Titer 96 Aqueous One Solution Reagent, Promega, Beijing, China). All experiments were performed triplicate.

### Cell migration and invasion assay

Twenty-four–well plates were applied with transwell chambers (corning) containing 8 μm polycarbonate membrane filters. For the invasion assay, diluted Matrigel (BD Biosciences) was used. Cancer cells (5 × 104/well) were planted in 200 μl of medium without FBS seeding on the upper chamber. The lower chamber was filled with 500 μl medium containing 15% FBS. After incubated for 24 h at 37 °C in 5% CO_2_, cells were fixed with 4% paraformaldehyde and the migrated cells were stained by 0.5% crystal violet (C8470, Solarbio, China), photographed and counted under a light microscope.

### Immunofluorescence

Cells of different groups were seeded and grown on glass slides at 24 h prior to the proper experiment. After fixation with 4% paraformaldehyde/PBS for 15 min, cells were washed once with PBS and permeabilized with 0.5% Triton X-100 for 10 min, and then blocked with 3% NGS (Gibco) for 30 min. The cells were then incubated with the anti-Arl13b (1:200, 17711-1-AP; Proteintech); anti-ac-tubulin (1:500, 66200-1-Ig; Proteintech); anti-SMO (1:200, sc166685; Santa Cruz) at 37 °C for 2 h, and then with a secondary antibody for 1 h at 37 °C. Nuclei staining was performed with 0.2 mg/mL DAPI.

### Immunohistochemistry

Immunohistochemical (IHC) staining was performed on the samples from the as previously described with antibodies specific for VHL, GLI1 and Ki-67. The degree of positivity was initially classified according to scoring both the proportion of positive staining tumor cells and the staining intensities. Scores representing the proportion of positively stained tumor cells were graded as: 0 (<10%); 1 (11–25%); 2 (26–50%); 3 (51–75%) and 4 (>75%). The intensity of staining was determined as: 0 (no staining); 1 (weak staining = light yellow); 2 (moderate staining = yellow brown); and 3 (strong staining = brown). The staining index was calculated as the product of staining intensity × percentage of positive tumor cells, resulting in scores of 0, 1, 2, 3, 4, 6, 8, 9 and 12. Only cells with clear tumor cell morphology were scored.

### Adenovirus transfection

LC3B-mCherry-GFP was delivered into adeno-associated virus and transfected into SN12-PM6 cell using Lipofectamine 2000 (Invitrogen). Quantification of fluorescence puncta was recorded and analyzed using fluorescence confocal microscope.

### Transmission electron microscope assay

SN12-PM6 cell was cultured with/without knockdown of IFT88 gene or treatment with Erismo before being trypsinized and fixed in 2.5% glutaraldehyde. Later, different cells were fixed in 1% osmium tetroxide with 0.1% potassium ferricyanide, dehydrated and embedded in epoxy resin. Samples were cut into ultrathin sections, which were stained with 2% uranyl acetate. The images were acquired using transmission electron microscope.

### Plasmid construction, transfection, and infection

Short hairpin RNA (shRNA) sequences targeting human IFT88, KIF3A, GLI1, VHL, oligonucleotides (IFT88: 5’-GAACAAGTTACAACTCCAGAA-3’; KIF3A: 5’-GCAACTAATATGAACGAACAT-3’; GLI1: 5’-CGTGAGCCTGAATCTGT-3’; VHL: 5’-TAGGATTGACATTCTTACAGTT-3’) were designed and synthesized by BGI (Shenzhen, China) and cloned into pLKO.1 vector. In addition, three mutation sites of PTCH1 (G37R, G466H, P1315L) and two mutation sites of PTCH2 (l104p, G207D) were constructed using whole plasmid, single-round PCR method. Two oligonucleotide primers containing the desired mutation sites were designed and synthesized, and are extended using DNA polymerase with wild-type plasmid template, and then a circular plasmid containing the indicated mutation site was obtained.

Lentivirus were generated in 293 T cells. Cells were transfected with 6 μg vector plasmids and 4.5 μg psPAX2 and 1.5 μg pMD2-VSVG using the standard calcium chloride transfection method. Calcium transfection kit was purchased from Macgene Biotech (Beijing, China). 48 h and 72 h after transfection, viral supernatant containing released viruses was collected and filtered through 0.45 μm filter. Target cells were infected with virus and 10 ug/ml polybrene for 24 h. Later, infected cells were selected with 2 μg/mL puromycin (Sigma, USA) for 3 or 4 days until they were stable. Knockdown of IFT88, KIF3A, GLI1 plasmids were transfected into SN12-PM6, Caki-1 and Caki-2 cells with 72 h.

### RNA extraction and qRT-PCR

TRIzol reagent (Invitrogen) was used to extract total RNA. Complementary DNA was synthesized with ProtoScript® II First-Strand cDNA Synthesis Kit (E6300S, NEB, USA) according to the manufacturer’s instruction. Afterwards, the mRNA expression levels of genes were detected with NovoStart® SYBR Green Super Mix Plus (E096-01A, NoVo protein, China). Relative mRNA expressions were normalized to peptidylprolyl isomerase A (PPIA) with the 2^−ΔΔCT^ method. The primer sequences used are listed in Supplementary Table S1.

### Primary renal cancer cell culture

Tumor specimens from two renal cell cancer patients were isolated with collagenase digestion and transported to the lab within 10 min. After removing blood clots, the samples were rinsed with sterile phosphate-buffered saline (PBS) twice and cut into small fragments in a size of about 1 mm^3^. Then, the fragments were incubated with collagenase of 1% (Solarbio) in a gently shaking water bath. After incubation with collagenase, the rest of samples were collected and put into 24 well plates. Cells were isolated and divided for culture using F medium containing 25% Ham’s F-12 nutrient mix (Thermo Fisher Scientific, Carlsbad, CA) supplemented with 10% FBS.

### In vivo metastasis studies

SN12-PM6 cells were prepared with Luciferase (for imaging), with/without knocked down of KIF3A, IFT88, GLI1. The cells (1 × 10^6^ in 0.1 mL of sterilized PBS) were mixed with Matrigel, 1:1 in respective groups were injected into the renal capsule of 4–5 week-old male nude mice (6 mice per group). The GLI1/2 inhibitor GANT61 (50 mg/kg) or DMSO was administered to the SN12-PM6-luc xenograft mouse models through an orogastric tub three times a week. Lesions, including the orthotopic xenograft tumors and lung metastatic nodules, were monitored in vivo with a molecular imaging system (NightOWL II LB983). The signal intensity of luc-labeled cells from lung tissues represented the amount of lung metastatic lesions. All mice were sacrificed 2 months post-injection. Tissue samples were collected and measured, and HE, IHC, IF staining were performed.

### Statistical analysis

The Cancer Genome Atlas (TCGA) database (http://cancergenome.nih.gov) was analyzed using UALCAN (http://ualcan.path.uab.edu/index.html) and Cbioportal database (https://www.cbioportal.org/) to determine Hedgehog, WNT and TGF-β signaling gene mutation. All analyses, unless specified otherwise, were performed using GraphPad Prism8.0.1 (GraphPad Software, CA, USA). Data are presented as the mean ± standard deviation (SD). Categorical data were analyzed with either chi-square or Fisher exact test. For comparison between two groups, unpaired student’s *t*-test was performed. The Kaplan–Meier survival analysis was performed using the log-rank test with the SPSS software (version 23.0). Significant differences were represented as *P < 0.05, **P < 0.01, ***P < 0.001, ****p < 0.0001 unless otherwise indicated.

## Results

### Primary cilia were preserved in VHL wild-type ccRCC and predict high risk of distal metastasis

**Fig. 1:**
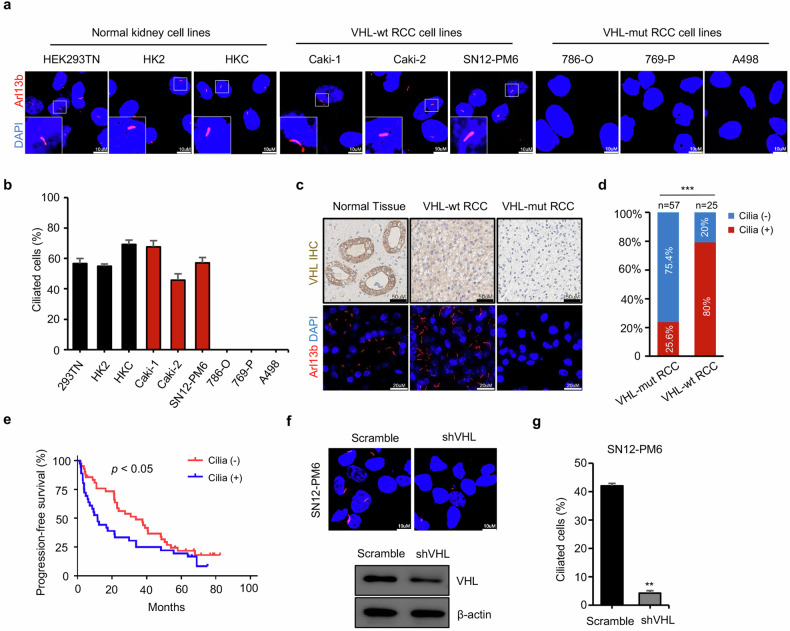
Primary cilia were preserved in VHL wild-type ccRCC and predicts high risk of distal metastasis. **a** Normal kidney cell lines (293TN, HK2, HKC), VHL wild type (VHL-wt) ccRCC cell lines (Caki-1, Caki-2, SN12-PM6) and VHL mutated (VHL-mut) ccRCC cell lines (786-O, 769-P, A498) were stained with antibodies for Arl13b (red) to mark cilia, and DAPI (blue) to mark DNA. Scale bar = 10 μm. **b** Quantification of ciliated cells in Normal kidney cell lines, VHL-wt ccRCC cell lines and VHL-mut ccRCC cell lines as shown in (**a**). *n* = 100. Error bars represent SD. **c** Representative IHC and IF staining in tissue samples. VHL-mut RCC, VHL-wt RCC and adjacent normal tissues were stained with antibodies for VHL (IHC), and Arl13b (red) to mark cilia, and DAPI (blue) to mark DNA. **d** Quantification of RCC samples with/without primary cilia in VHL-mut RCC group (*n* = 57) and VHL-wt RCC group (*n* = 25), *P* < 0.05, Fisher’s exact test. **e** Kaplan–Meier analysis indicated that ccRCC with primary cilia was associated poor progression free survival (*P* < 0.05). **f** Immunofluorescence staining showed the loss of cilia in SN12-PM6 induced by VHL knockdown. Primary cilia were marked with Arl13b (red) and DNA was marked with DAPI (blue). Scale bar, 10 μm. Western blot assay showed efficiency of VHL knockdown in SN12-PM6 cell line. **G** Quantification of experiment shown in (**f**). Error bars represent SD. **P < 0.01.

ccRCC is always accompanied with the VHL inactivation and concurrent loss of primary cilia, which were thought to act as suppressors in tumor progression [2, 3, 25]. However, the extent of ciliation and its role in the VHL wild-type ccRCC remains largely unknown. To determine whether ciliogenesis is associated with the VHL mutation status, we stained various renal cell lines with cilia marker Arl13b. Indeed, normal kidney cell lines were ciliated, while VHL mutated (VHL-mut) ccRCC cell lines were mostly devoid of primary cilia (Fig. 1a, b, Supplementary Fig. 1A), in line with previous studies [26]. In contrast, VHL wild-type (VHL-wt) ccRCC cell lines preserved high incidence of primary cilia, with a frequency of ciliated cells between 40% to 70% (Fig. 1a, b). To further reinforce the link between ciliation and the VHL status, we reverted to clinical ccRCC tumor samples. We performed VHL genetic mutation analysis using sanger sequencing in 82 patients, of which 25 cases had no VHL genetic mutation. Then these tumor samples and adjacent normal tissues underwent immunohistochemistry (IHC) staining with antibody for VHL and immunofluorescence (IF) staining with cilia marker Arl13b (Fig. 1c). Compared to VHL-mut ccRCCs, VHL-wt ccRCCs had high expression levels of VHL (Fig. 1c) and maintained high incidence of primary cilia (Fig. 1c, d), indicating that the VHL status correlated positively with the presence of cilia. Furthermore, prognostic analysis revealed that primary cilia are associated with poor progression-free survival (PFS) (Fig. 1e) but not with overall survival (Supplementary Fig. 1B). To test whether VHL regulated primary cilium formation, we established a VHL-knock-down renal cancer cell line (SN12-PM6) using stable RNA interference (Fig. 1f) and analyzed the cilia frequency. The results demonstrated that knockdown of VHL significantly decreased ciliation in SN12-PM6 (Fig. 1g).

Taken together, these findings showed that primary cilia were kept extensively in VHL-wt ccRCC and its presence predicted a significantly worse PFS in ccRCC.

### Inhibition of ciliogenesis suppresses VHL wild-type ccRCC growth and metastasis

**Fig. 2:**
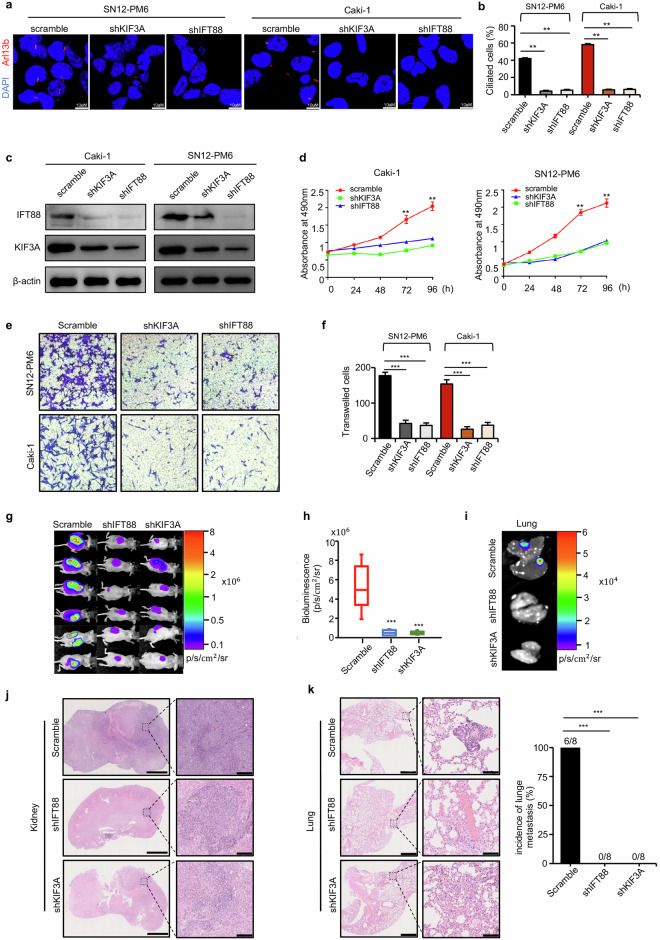
Inhibition of ciliogenesis suppresses VHL wild-type ccRCC growth and metastasis. **a** Immunofluorescence staining with antibodies Arl13b (red) to mark cilia and DAPI (blue) to mark DNA after lentiviral transduction of shIFT88 or shKIF3A showed inhibition of primary cilia formation in ccRCC (Caki-1, SN12-PM6) cell lines. Scale bar = 10 μm. **b** Quantification of percentage of Caki-1, SN12-PM6 cells with primary cilia after stable silencing of IFT88 and KIF3A. **c** Western blot assay showed IFT88 and KIF3A protein levels with IFT88 and KIF3A knockdown in Caki-1, SN12-PM6 cells. **d** MTT assays showed that loss of primary cilia induced by IFT88 and KIF3A knockdown reduced the proliferation velocity in Caki-1, SN12-PM6. **e** Transwell assays showed that loss of primary cilia induced by IFT88 and KIF3A knockdown reduced the migration and invasion in Caki-1, SN12-PM6. **f** Quantification of transwelled cells as shown in (**e**). **g** Representative bioluminescent images of nude mice that underwent orthotopic implantation with Luc-labeling SN12-PM6 cells stably transfected by shKIF3A, shIFT88 or scramble vectors in 4 weeks. **h** Measurement of bioluminescent signals of experiments shown in (**g**). **i** Representative bioluminescent images of lung tissues in each group as shown in (**g**). **j** Representative H&E staining of kidney in each group as shown in (**g**). Scale bar (left panel) = 2 mm, scale bar (right panel) = 50 μm. **k** Representative H&E staining of lung tissue in each group as shown in (**g**). Scale bar (left panel) = 1 mm, scale bar (right panel) = 100 μm. The statistical graph indicated the incidence of lung metastasis in each group. In all panels, *P < 0.05, **P < 0.01, ***P < 0.001.

As VHL-wt ccRCC cell lines Caki-1 and SN12-PM6 with metastatic potential harbored high incidence of primary cilia and clinical patients with ciliation were more likely to experience relapse and metastasis, we proposed a novel hypothesis that primary cilia may have a tumor-promoting effect in VHL-wt ccRCC. We then assessed whether cilia deconstruction mediated by ciliary gene suppression affects renal cancer cell behavior. We found silencing of KIF3A and IFT88 in Caki-1 and SN12-PM6 significantly blocked ciliogenesis (Fig. 2a, b) and the knockdown efficiency was confirmed at protein levels by western blot (Fig. 2c). The effect of ciliary genes on ccRCC cell proliferation was then examined using MTT assay. The results showed that inhibition of ciliogenesis significantly reduced cell proliferation in both Caki-1 and SN12-PM6 cells (Fig. 2d). Further transwell assay showed that ciliary genes knockdown strongly decreased the migrated ccRCC cell number when compared to the scramble controls (Fig. 2e, f). Next, we tested whether inhibition of ciliogenesis would impact the tumorigenic and metastasis capacity of ccRCC cells in vivo. As we previously proved that SN12-PM6 cells could effectively develop tumors and metastasis in vivo [27–29], luciferase labeled SN12-PM6 cells were transfected with scramble, shIFT88, and shKIF3A respectively and orthotopically implanted into the sub-renal capsule of mouse kidney. After 4 weeks, bioluminescent signals in shIFT88 and shKIF3A groups were significantly lower than scramble group (Fig. 2g, h). Then we performed ex vivo bioluminescent imaging immediately after mice were killed to monitor the lung metastases, and the results showed that pulmonary metastases could be developed in scramble group but not in shIFT88 or shKIF3A group (Fig. 2i). H&E staining (Fig. 2j) of the kidneys confirmed the ccRCC tumor type and demonstrated that tumor volumes of shIFT88 and shKIF3A groups were significantly decreased compared to scramble group. Furthermore, H&E staining of lung tissues confirmed pulmonary metastases in scramble group but not in shIFT88 or shKIF3A group (Fig. 2k). To further validate the knockdown efficiency in vivo, we examined the cilia in animal samples with Arl13b immunostaining. The results confirmed that knockdown of KIF3A and IFT88 significantly inhibited ciliogenesis in tumor tissues (Supplementary Fig. 2A).

Taken together, these findings demonstrated that primary cilia play an oncogenic role in VHL wild-type ccRCC proliferation and metastasis both in vitro and in vivo.

### Hedgehog pathway mutations are enriched in VHL wild-type ccRCC and encode pathogenic alleles

**Fig. 3:**
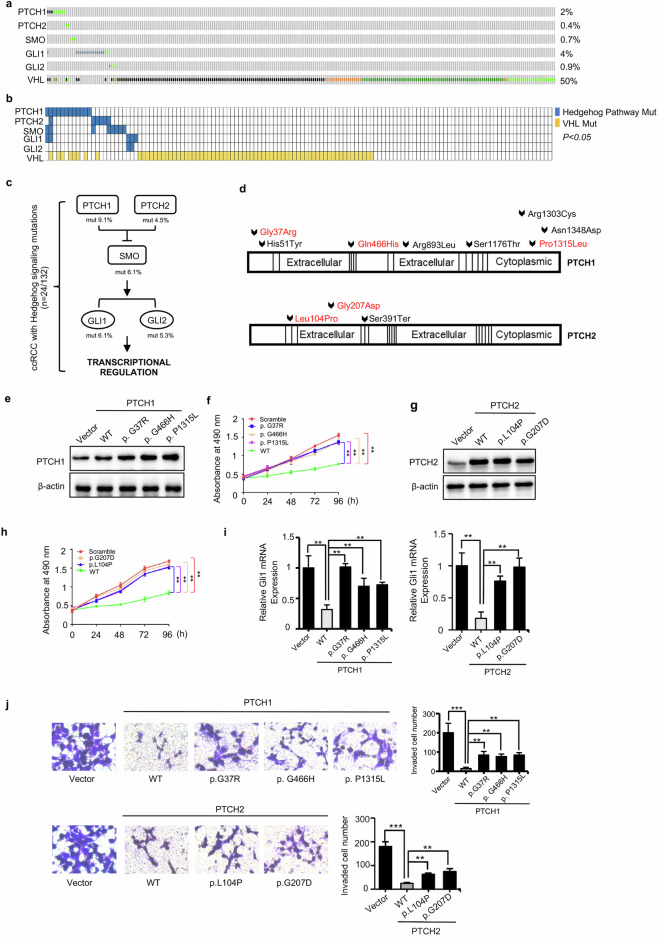
Hedgehog pathway mutations are enriched in VHL wild-type ccRCC and encodes pathogenic alleles. **a** Frequency of gene mutations in hedgehog signaling pathway and VHL in renal cancer based on TCGA database. **b**.Targeted exon sequencing data from ccRCC patients in our center (*n* = 132) showed mutations in hedgehog signaling and the correlation between hedgehog pathway genes mutations and VHL mutation, *P* < 0.05. **c** The frequency of mutated genes of hedgehog signaling pathway from the targeted exon sequencing data in our center. **d** Amino acid alterations predicted to be most significantly affected of PTCH1 and PTCH2 were annotated on the transcriptional variant. **e** Mutations of PTCH1 as indicted were constructed and transduced into SN12-PM6. Western blot assay showed the protein expression level of PTCH1 for each group. **f** MTT assays showed that mutated PTCH1 lost inhibition of the proliferation velocity in SN12-PM6, compared to wild-type PTCH1. **g** Mutations of PTCH2 as indicted were constructed and transduced into SN12-PM6. Western blot assay showed the protein expression level of PTCH2 for each group. **h** MTT assays showed that mutated PTCH2 lost inhibition of the proliferation velocity in SN12-PM6, compared to wild-type PTCH2. **i** Relative GLI1 mRNA expression in overexpression of wild-type and mutated PTCH1 and PTCH2 in SN12-PM6. **j** Transwell assays showed that overexpression of mutated PTCH1 and PTCH2 lost inhibition of the migration and invasion of SN12-PM6, compared to wild-type PTCH1 and PTCH2. In all panels, *P < 0.05, **P < 0.01, ***P < 0.001.

Several signaling pathways important for tumor progression have been associated with the primary cilium, especially the well-characterized Hedgehog pathway. Through comprehensive big data analysis, we pointed out that Hedgehog signaling pathway possesses higher mutation frequency (7.6%, *n* = 34 of 448) compared to Wnt (1.2%) and TGF-β (0.4%) pathways (Fig. 3a and Supplementary Fig. 3A). Moreover, Hedgehog pathway mutations are more prevalent in VHL wild-type patients. Among the 34 patients who have hedgehog signaling mutations, 22 patients are VHL wild type and 12 patients are VHL mutant (Supplementary Fig. 3B). To further validate this phenomenon, we integrated the targeted exon sequencing data from renal cancer patients in our hospital and found mutations of genes of Hedgehog pathway accounted for 18% (*n* = 24 of 132) (Fig. 3b, c). Additionally, 16 patients are VHL wild type with hedgehog signaling mutations and only 8 patients are VHL mutant, indicating that Hedgehog signaling may be crucial for the malignant progression of VHL-wt ccRCC (Supplementary Fig. 3B).

Loss-of-function mutations of PTCH1/2 result in aberrant activation of Hedgehog signal pathway, and are driver oncogenic mutations in some tumor types, including basal cell carcinoma, medulloblastoma and rhabdomyosarcoma [30–34]. To evaluate the function of these mutations, we constructed three mutation sites of PTCH1 and two mutation sites of PTCH2, which were predicted to mutate evolutionarily conserved amino acid residues (Fig. 3d), and then transduced the wild-type PTCH1/2 and their mutants in SN12-PM6 cells. We found that the mutant PTCH1/2 alleles tested had impaired growth-suppressive activity (Fig. 3e–h) and migration-suppressive activity when compared to wild-type PTCH1/2 (Fig. 3j), with a concordant impairment in their ability to suppress hedgehog signaling, as assessed by GLI1 mRNA expression (Fig. 3i).

Taken together, these findings illustrate the hedgehog pathway mutations were significantly associated with VHL-wt ccRCC and the tested PTCH1/2 mutations encode functionally impaired alleles.

### Primary cilia are required for hedgehog signaling activation

**Fig. 4:**
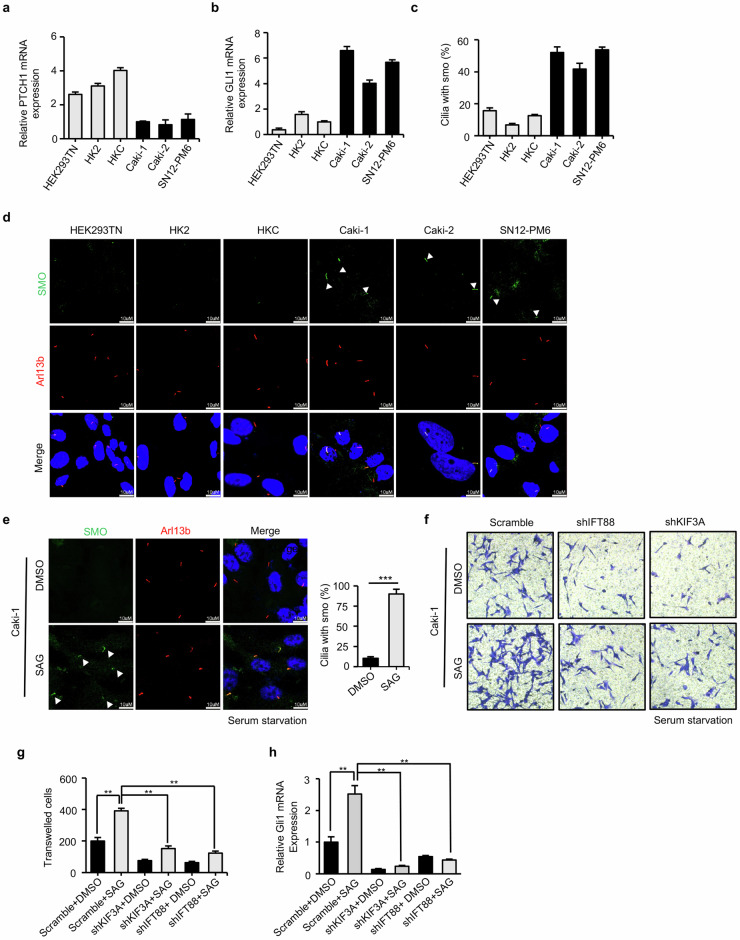
Primary cilia are required for hedgehog signaling activation. **a**, **b** qPCR showed mRNA levels of Hedgehog pathway target genes of PTCH1 (**a**) and GLI1 (**b**) in ciliated ccRCC cell lines (Caki-1, Caki-2, SN12-PM6) and normal kidney cell lines (293TN, HK2, HKC). **c** Quantification of cilia with SMO localization as experiment shown in (**d**). Error bars represent SD. **P < 0.01. **d** Immunofluorescence staining with antibodies for Arl13b (red), SMO (green) and DAPI (blue) in ciliated ccRCC cell lines (Caki-1, Caki-2, SN12-PM6) and normal kidney cell lines (293TN, HK2, HKC) demonstrated SMO ciliary localization. White arrows indicated SMO in cilia. **e** Caki-1 was starved for 48 h and treated with DMSO or SAG (1 μM), and then fixed and stained with antibodies for Arl13b (red), SMO (green), and DAPI (blue). Note that starvation reduced SMO ciliary localization while SAG (1 μM) treatment promoted SMO ciliary localization. White arrows indicated SMO in cilia. **f** Caki-1 cells were transfected with shIFT88, shKIF3A for 72 h, and then starvation for additional 48 h and treated with DMSO or SAG (1 μM). Transwell assays showed that migration and invasion in scramble group were significantly promoted by SAG treatment but not in shIFT88 or shKIF3A group (loss of primary cilia). **g** Quantification of transwelled cells as shown in (**e**). **h** qPCR showed mRNA levels of GLI1 by indicated treatments. In all panels, *P < 0.05, **P < 0.01, ***P < 0.001.

As we had proved that Hedgehog pathway mutations were enriched in VHL-wt ccRCC, we wondered whether hedgehog signaling is activated in VHL-wt ccRCC cell lines and its activation depends on primary cilia. Firstly, we performed quantitative real-time PCR (qPCR) to detect mRNA levels of Hedgehog target genes of PTCH1 and GLI1 in VHL-wt ccRCC cell lines and in normal kidney cell lines. The results showed that mRNA levels of PTCH1, an inhibitor of Hedgehog pathway, were significantly lower in VHL-wt ccRCC cell lines while mRNA levels of transcription factor GLI1, a terminal effector of the Hedgehog pathway, were significantly higher in VHL-wt ccRCC cell lines compared to normal kidney cell lines (Fig. 4a, b). As a key step of Hedgehog signaling activation is smoothened (SMO) protein accumulating in cilia, the results from double immunostaining showed that SMO ciliary localization in VHL-wt ccRCC cell lines were more than normal kidney cell lines (Fig. 4c). These above data suggested that hedgehog signaling was activated in VHL-wt ccRCC. To functionally link hedgehog signaling activation to primary cilia formation, we relied on serum-free cell culture with low baseline SMO ciliary localization but strong induction upon SAG treatment, a highly potent agonist of SMO to activate hedgehog pathway. (Fig. 4d). Under serum starvation conditions, transwell assays found that SAG treatment could significantly promote migration of Caki-1 in scramble group, but not in groups with suppression of ciliary genes (Fig. 4e-g). Furthermore, primary cilia deconstruction compromised GLI1 signaling activated by SAG treatment (Fig. 4h).

Thus, these findings demonstrated an essential role for primary cilia as a platform for the activation of the Hedgehog signaling pathway in VHL-wt ccRCC.

### Targeting hedgehog signaling restrains VHL wild-type ccRCC malignant progression

**Fig. 5:**
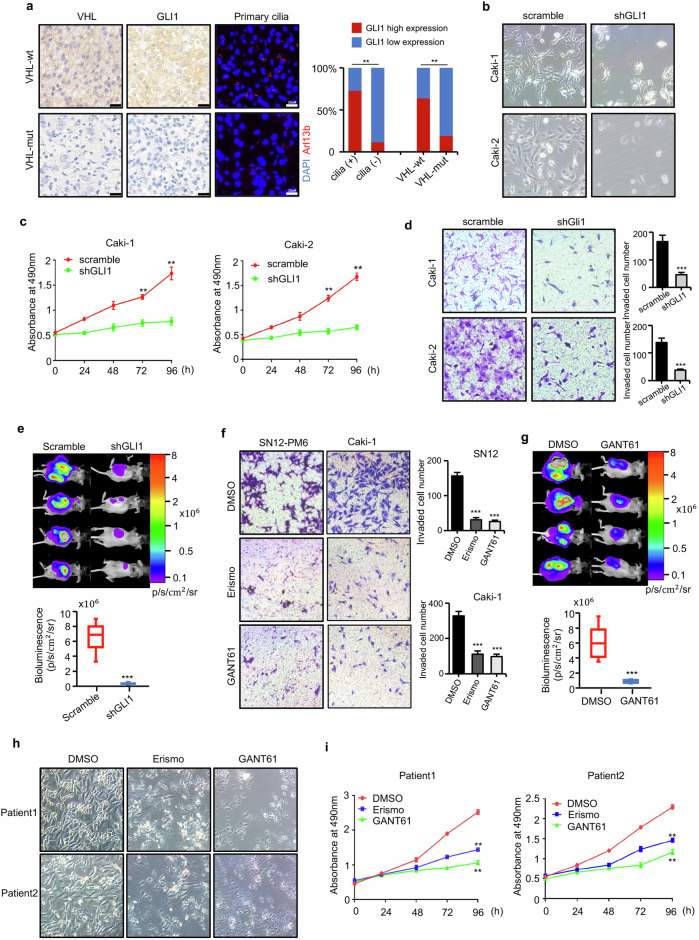
Targeting hedgehog signaling restrains VHL wild-type ccRCC malignant progression. **a** IHC assays and immunofluorescence staining were performed in clinical ccRCC samples (*n* = 49) to demonstrate the positive correlation between expression of VHL, GLI1, and primary cilia. Scale bars = 20 μm. **b** Cell numbers and morphology depicted the effect of knockdown of GLI1 in ccRCC cells (Caki-1, Caki-2). **c** MTT assays showed that knockdown of GLI1 significantly inhibited the proliferation velocity of Caki-1 and Caki-2. **d** Transwell assays showed that knockdown of GLI1 significantly inhibited migration and invasion of Caki-1 and Caki-2. **e** Representative bioluminescent images of nude mice that underwent orthotopic implantation with Luc-labeling SN12-PM6 cells stably transfected by shGLI1 and scramble vectors in 4 weeks. Measurement of bioluminescent signals of each group was shown in lower panel. **f** Transwell assays showed that inhibition of SMO by Erismodegib (10 μΜ) or inhibition of GLI1 by GANT61 (5 μΜ) could significantly inhibited migration and invasion of SN12-PM6 and Caki-1 cells. **g** Representative bioluminescent images of nude mice that underwent orthotopic implantation with Luc-labeling SN12-PM6 cells and oral treatment of GANT61 (50 mg/kg) or DMSO in 4 weeks. Measurement of bioluminescent signals of each group was shown in lower panel. **h** Cell numbers and morphology depicted the effect of Erismodegib (10 μΜ) and GANT61 (5 μΜ) on primary cells derived from two human ccRCC samples. **i** MTT assays showed that Erismodegib (10 μΜ) and GANT61 (5 μΜ) significantly inhibited the proliferation velocity of primary cells derived from human ccRCC samples.

Previous studies have suggested that the sonic hedgehog signaling pathway can be reactivated by PI3K/AKT signaling in human renal cell carcinoma independently of VHL gene status, but its role in VHL-wt ccRCC has not been examined. Firstly, we analyzed TCGA database showing that expression of GLI1 is higher in ccRCC tumors than in normal tissue and high expression of GLI1 is associated with poor survival (Supplementary Fig. 4A, B). Next, we performed immunostaining in clinical specimens indicating that high GLI1 expression was mostly detected in VHL-wt ccRCC and increased GLI1 protein correlated positively with ciliary formation (Fig. 5a). In addition, western blot assays also demonstrated that the GLI1 protein level is higher in VHL-wt ccRCC cell lines than VHL-mutated ccRCC cell lines (Supplementary Fig. 4C). Then, we knockdown GLI1 in Caki-1 and Caki-2 cells and functional studies were performed (Supplementary Fig. 4D, Fig. 5b). Downregulation of GLI1 significantly inhibited cell proliferation and migration (Fig. 5c, d). To confirm the suppression phenotype of GLI1 depletion in vivo, SN12-PM6 stably expressing luciferase with knockdown of GLI1 was implanted orthotopically into the right sub-renal capsule of nude mice. After 4 weeks, bioluminescent imaging showed that the signals in the kidneys were significantly higher in control group than those in shGLI1 group (Fig. 5e). IHC staining confirmed the decreased expression of Ki67 in xenograft tumors with GLI1 silencing (Supplementary Fig. 4E).

Hedgehog pathway inhibitors have shown great efficacy in treating hedgehog-mediated malignancies, including basal cell cancer and medulloblastoma. Then we used the Erismodegib (inhibitor of SMO) and GANT61 (inhibitor of GLI1) to test their role in VHL-wt ccRCC cells in vitro and in vivo. Results showed that these two drugs dramatically inhibited the proliferation and migration of SN12-PM6 cells (Fig. 5f, Supplementary Fig. 4F, G). To confirm the suppression effect of hedgehog pathway inhibitors in vivo, GANT61 (50 mg/kg) or DMSO was administered to the SN12-PM6-luc xenograft mouse models through an orogastric tub three times a week. After 4 weeks, bioluminescent imaging showed that the signals in the kidneys were significantly lower in GANT61-treated group than the control group (Fig. 5g).

Furthermore, we established primary tumor cells with considerable ciliation from two VHL-wt ccRCC patients (Supplementary Fig. 4H). Similarly, blocking hedgehog signaling with inhibitors significantly suppressed the primary ccRCC cell growth (Fig. 5h, i). Collectively, our findings demonstrated the dependency of hedgehog signaling for the malignant progression of VHL-wt ccRCC.

### Inhibition primary cilia-hedgehog signaling axis provokes autophagic cell death

**Fig. 6:**
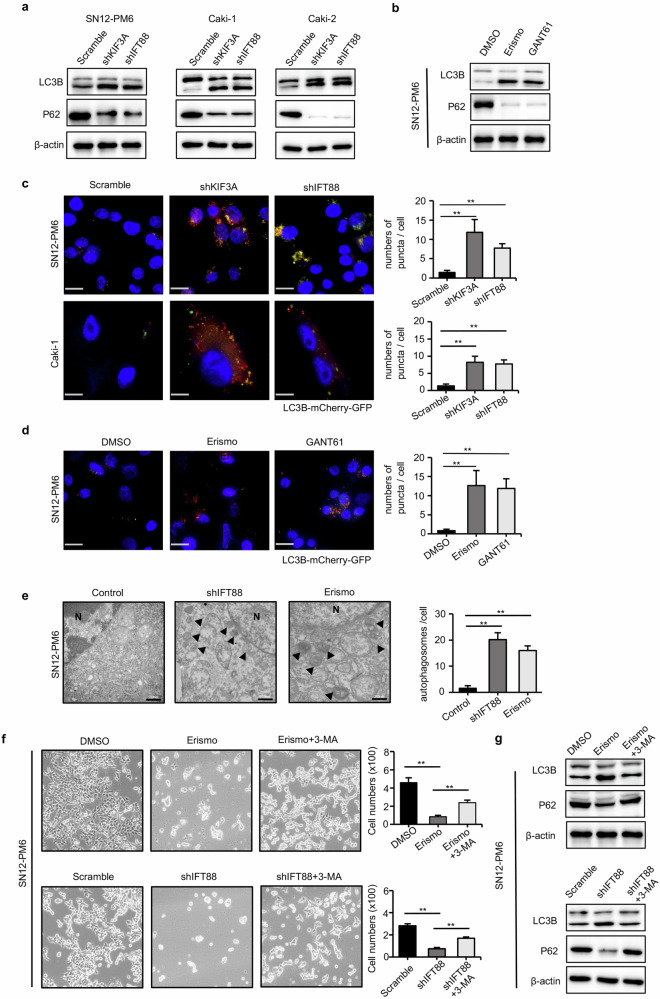
Inhibition primary cilia-hedgehog signaling axis provokes autophagic cell death. **a** Western blot assays showed the expression level of LC3B and P62 in SN12-PM6, Caki-1, Caki-2 cells with knockdown of KIF3A, IFT88. **b** Western blot assays showed the expression level of LC3B and P62 in SN12-PM6 with treatment of Erismodegib (10 μΜ), GANT61 (5 μΜ) or DMSO. **c** SN12-PM6, Caki-1 cells were transfected with mCherry-GFP-LC3B. After knockdown of KIF3A and IFT88, representative images of LC3-positive puncta were obtained with a confocal fluorescence microscope. Quantification of puncta numbers of each group was shown in right panel. Error bars represent SD. Scale bars = 10 μm. **d** SN12-PM6 Cells were transfected with mCherry-GFP-LC3B and treated with Erismodegib (10 μΜ) or GANT61 (5 μΜ), and LC3 -positive puncta were obtained. Quantification of puncta numbers of each group was shown in right panel. Error bars represent SD. Scale bars = 10 μm. **e** Transmission electron microscope figures showed that autophagosomes in the cytoplasm were increased in SN12-PM6 after knockdown of shIFT88 or treated with Erismodegib (10 μΜ). Quantification of puncta numbers of each group was shown in right panel. Error bars represent SD. Scale bars = 500 nm. **f** Cell numbers and morphology showed the effect of inhibitor of autophagy flux (3MA) in SN12-PM6 with knockdown of shIFT88 or treated with Erismodegib (10 μΜ). **g** Western blot assays showed the effect of 3MA on the expression level of LC3B and P62 in SN12-PM6 with knockdown of shIFT88 or treated with Erismodegib (10 μΜ).

Many studies have suggested that primary cilia and the Hedgehog signaling from the cilia can regulate autophagic activity, which may affect tumor progression through a cell survival or cell death mechanism, depending on the cellular status. To determine the effect of primary cilia-hedgehog signaling axis on autophagy, we investigated the level of LC3B, the number of autophagic puncta and the formation of autophagosomes in VHL-wt ccRCC cells. Western blotting results revealed that ciliary defects or treatment with Hedgehog signaling inhibitors increased the turnover of the autophagic marker LC3B and P62 degradation (Fig. 6a, b), In accordance with these results, the number of GFP-mCherry-LC3-II-positive puncta was significantly higher after inhibition of primary cilia-hedgehog signaling axis than that in the control cells (Fig. 6c, d). The TEM results also indicated that inhibition of ciliogenesis or blocking hedgehog signaling induced more autophagosomes and autolysosomes in the cytoplasm than in the control cells (Fig. 6e). These data indicated that blocking the primary cilia-hedgehog signaling axis enhanced the autophagic flux.

Since inhibition of primary cilia-hedgehog signaling axis led to significant cell death, we wondered whether autophagy plays a pro-death role during the process. Ciliary gene knockdown or Erismodegib treatment combined with the autophagy inhibitor 3-MA greatly prevented the cell death (Fig. 6f). Western blotting results also revealed that treatment of 3-MA in SN12-PM6 with ciliary defects or hedgehog signaling inhibitor significantly inhibited autophagic marker LC3B and SQSTM1 degradation (Fig. 6g). We also examined the influence of Chloroquine (CQ) on autophagic cell death caused by cilia-Hh inhibition. The results indicated that CQ could largely rescue cell death in common with 3-MA (Supplementary Fig. 5A). Besides, we found that CQ treatment moderately increases the autophagosome puncta and the percentage of ciliated cells (Supplementary Fig. 5B, C). Since AMPK is a key energy sensor that initiates autophagy [35, 36], we detected AMPK’s activity in GANT61-treated ccRCC cells. Our results elucidated that GANT61 activated the AMPK signaling pathway, and pharmacological inhibition of AMPK with compound C could inhibit autophagy and partially rescue GANT61-induced autophagic cell death (Supplementary Fig. 5D–F). Furthermore, tyrosine kinase inhibitor (TKI) such as sunitinib, has been reported to induce autophagic cell death in ccRCC [37]. We detected the synergistic effect of GANT61 on cell growth suppression and found that GANT61 could enhance sunitinib efficiency via inducing autophagic cell death (Supplementary Fig. 6A, B). Future studies using mouse models to explore in vivo synergistic function on tumor suppression are required.

These results strongly indicate that autophagy serves as the predominant method by which inhibition primary cilia-hedgehog signaling axis induces cell death.

## Discussion

Many studies from different groups have reported that patients with wild-type VHL (VHL-wt) exhibit worse outcomes than those with inactivated VHL [38–40], but the underlying mechanisms are poorly understood. In this study, we detected primary cilia by immunofluorescence analysis both in ccRCC cell lines and clinical specimens, finding that VHL-wt ccRCC preserved high incidence of ciliation and its presence predicted poor prognosis. These data suggest that primary cilia can be an independent prognostic factor for recurrence in ccRCC. An independent study also found that GLI1/IFT20 signature can predict the presence of primary cilia and is positively correlated with poor prognosis [41], further supporting our findings.

VHL also exerts HIF-independent functions, especially in regulation of primary cilium. Many studies have reported that VHL overexpression can restore ciliogenesis in VHL-mut ccRCC cell lines [26, 42]. However, whether VHL knockdown or knockout affects ciliation is contradictory. Previous studies found that deletion of VHL in primary MEFs had no effect on cilia morphology and number, while silencing of VHL in hTERT RPE-1 results in decrease and shortening of primary cilia [15]. In our study, we not only explored the correlation of VHL status with cilia formation, but also investigated VHL-mediated cilia function in VHL-wt ccRCC. We found that VHL knockdown could lead to reduced cilia frequency in VHL-wt ccRCC cells, implicating an indispensable role for VHL in maintaining ciliogenesis in renal cancer cells.

ccRCC was characterized by significantly reduced frequency of primary cilia both in human tumor samples [25] and mouse-derived tumor models [13]. Besides, loss of primary cilia can drive renal cyst growth and renal carcinogenesis [15, 16, 43, 44], demonstrating a tumor-suppressive role of primary cilia in ccRCC. However, almost all these studies were based on the model with VHL gene mutation, and specific function of primary cilia in VHL-wt ccRCC was poorly studied. Interestingly, VHL-wt cell lines (Caki-1 and SN12-PM6) with high incidence of primary cilia possess great invasive and metastatic capacity. Moreover, several studies also illustrated that primary cilia have a dual and opposing role that can both mediate or suppress tumorigenesis even in the same tumor type depending on oncogenic process [7, 8]. This evidence reminds us that primary cilia may play an oncogenic role in progression of VHL-wt ccRCC. Functional studies further confirmed that inhibition of ciliogenesis could significantly suppress VHL-wt ccRCC growth and metastasis both in vitro and in vivo. Thus, we uncovered a dual and opposing role of primary cilia in different ccRCC subtypes according to the VHL status.

Hedgehog (Hh) signaling, which is functionally linked to primary cilia, has been shown to participate in tumorigenesis and tumor progression [45, 46]. By analyzing the TCGA ccRCC dataset and targeted exon sequencing data from renal cancer patients of our hospital, we have identified a high frequency of Hh pathway mutations, accounting for 8.26% and 18% respectively. Surprisingly, compared to VHL-mut ccRCC, mutations of Hedgehog pathway were more enriched in VHL-wt ccRCC. Functional studies show that mutant PTCH1/2 alleles had impaired tumor-suppressive activity when compared to wild-type PTCH1/2, owing to the downstream overactivation of Hh signaling. Besides, we also found SMO accumulated in the ciliary membrane, and GLI was highly expressed in VHL-wt ccRCC tissues and positively correlated with ciliation. Moreover, inhibition of ciliogenesis significantly blocked Hh signaling activation. This evidence further strengthened the functional connection between cilia and Hh signal transduction in VHL-wt ccRCC. Though activations of Hh signaling have been associated with poor progression of ccRCC [47–49], we firstly provide evidence that Hh pathway activation in VHL-wt ccRCC depends on the primary cilia and VHL-wt ccRCC is more susceptible to Hh inhibitors than VHL-mut ccRCC, suggesting that targeting Hh signaling may be an attractive therapeutic strategy for VHL-wt ccRCC.

Autophagy is in general considered an important homeostatic mechanism that acts as double-edged sword for cancer in both proliferation and metastasis depending on the cancer subtype [50]. Everolimus, an autophagy inducer, represents a therapeutic option for the treatment of advanced RCC [51]. Previous studies also indicated that upregulated autophagy could suppress proliferation and cause cell death of RCC cells [52, 53]. Since both primary cilia and Hh signaling are involved in regulation of autophagy, we detected the autophagic flux by investigating the level of LC3B, the number of autophagic puncta and the formation of autophagosomes in VHL-wt ccRCC cells. Our research demonstrated that inhibition of ciliogenesis or blocking hedgehog signaling could activate autophagy and induce autophagic cell death which can be greatly alleviated by autophagy inhibitor 3-MA and CQ. Besides, AMPK is an upstream kinase to initiate autophagy, we found that GANT61 treatment could activate AMPK signaling and AMPK inhibitor compound C could also suppress autophagy and partially rescue GANT61-induced autophagic cell death. Functional interplay between primary cilia and autophagy is not well understood in ccRCC, our data illustrated that primary cilia is required for maintaining low level of autophagy to promote VHL-wt ccRCC progression. Whether hedgehog signaling is involved in the molecular mechanisms of cilia-related autophagy needs further studies.

In summary, our study demonstrates that primary cilia serve as a functional platform to activate Hh signaling and inhibit autophagy, providing a novel insight into the mechanism of malignant progression for VHL-wt ccRCC. Targeting primary cilia-hedgehog signaling axis may provide promising therapeutic targets for advanced VHL-wt ccRCC.

## Supplementary information

supplementary figures1-6

supplementary table

Original data

## Data Availability

The data used to support the findings of this study are available from the corresponding author upon request. And the data that support the first part of findings in this study are openly available in TCGA database.
