# Supplementary material for: Inhibition of primary cilia-hedgehog signaling axis triggers autophagic cell death and suppresses malignant progression of VHL wild-type ccRCC
Source: Cell Death Dis. 2024 Oct 10;15(10):739. doi: 10.1038/s41419-024-07085-8 (PMC11466958; doi:10.1038/s41419-024-07085-8)
Supplement: Supplementary file 1 — supplementary figures1-6 [file 41419_2024_7085_MOESM1_ESM.pdf]

Supplementary Materials

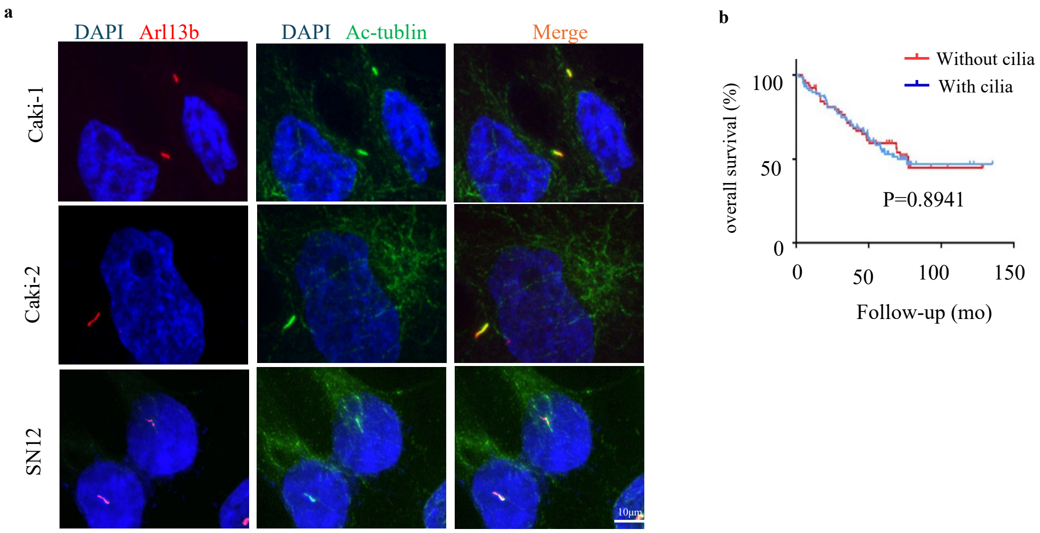

Figure S1 Primary cilia expression in VHL-wt ccRCC and correlation with overall survival.

(**a**) VHL wild type (VHL-wt) ccRCC cell lines (Caki-1, Caki-2, SN12-PM6) were double-stained with antibodies for Arl13b (red) and Ac-tublin to mark cilia, and Hoechst 33342 (blue) to mark DNA. Scale bar = 10 μm. (**b**) Kaplan-Meier analysis indicated that there was no significant difference between ccRCC with primary cilia or without primary cilia. ($P > 0.05$).

a

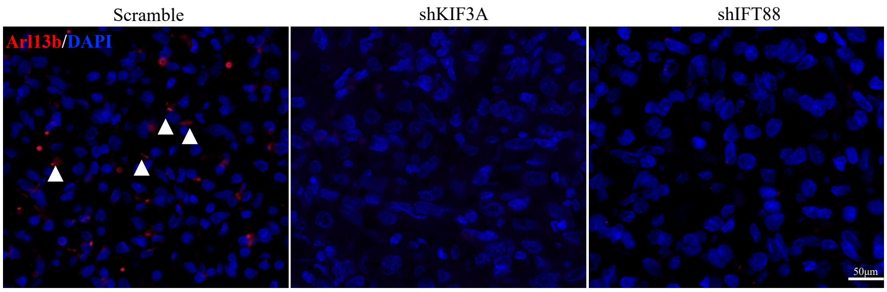

Figure S2 Knockdown of KIF3A and IFT88 inhibits primary cilia formation.

(**a**) Representative images of immunofluorescence staining with antibodies Arl13b (red) to mark cilia and Hoechst 33342 (blue) to mark nuclei after lentiviral transduction of shIFT88 or shKIF3A in ccRCC tumor samples. Scale bar = 50 μm.

a

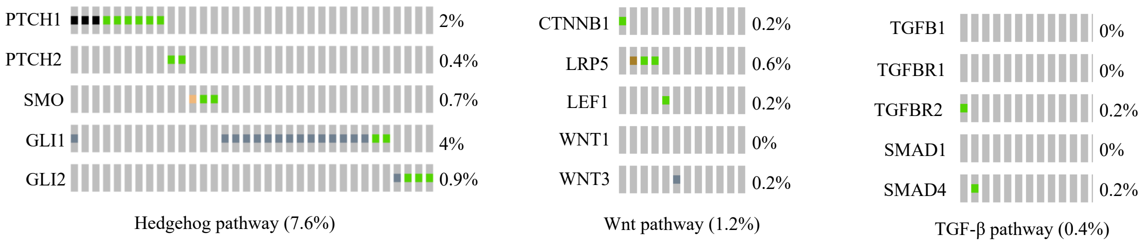

b

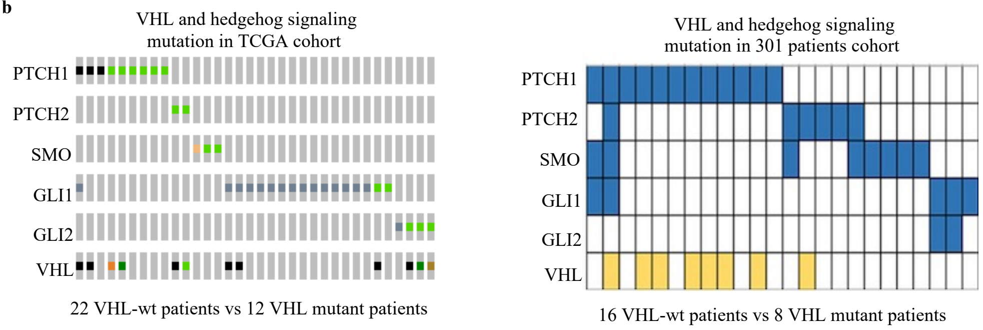

Figure S3 Mutations in the Hedgehog signaling pathway are prevalent in VHL wild-type ccRCC.

(**a**) Frequency of gene mutations in Hedgehog signaling pathway, Wnt and TGF-β pathways in ccRCC based on TCGA database (open source http://www.cbioportal.org/). (**b**) The rate of Hedgehog signaling pathway mutation in VHL-wt ccRCC in TCGA cohort and 301 patients cohort.

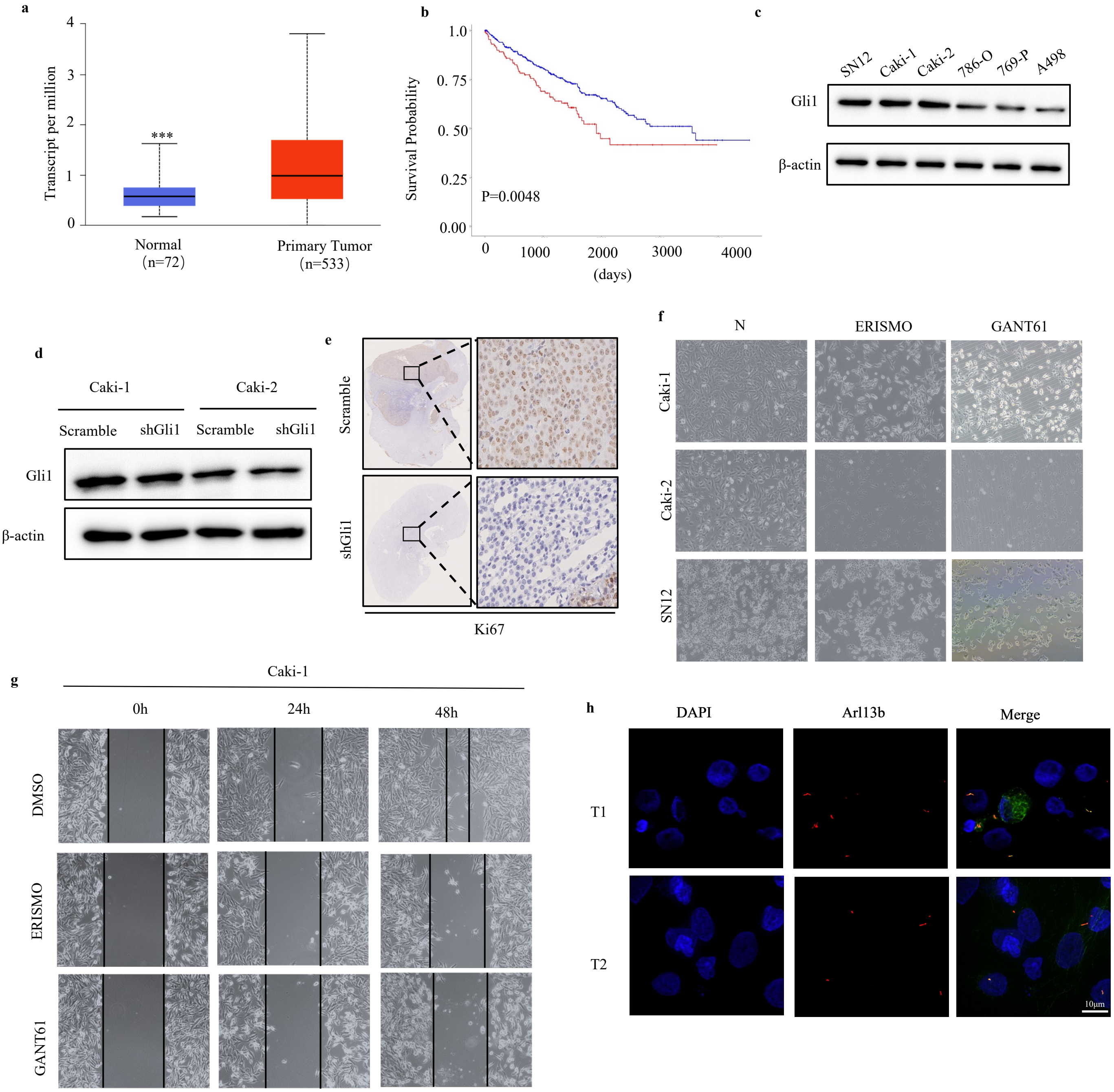

Figure S4 Inhibition of Hedgehog signalling pathway suppresses ccRCC.

(**a**) TCGA database analyze showed expression of GLI1 is higher in tumor than normal tissue, p<0.01. (**b**) TCGA database analyze showed high expression of GLI1 is associated with poor survival, p<0.01. (**c**) Western blot assay showed GLI1 protein levels in VHL wild-type cell lines Caki-1, Caki-2, SN12-PM6 cells and VHL mutated cell lines 786-O, 769-P, A498. (**d**) Western blot assay showed GLI1 protein levels in Caki-1 and Caki-2 with knockdown of GLI1. (**e**) IHC staining showed the level ki67 in the tumor of nude mice. (**f**) Cell numbers and morphology depicted the effect of GLI1 inhibitors Erismodegib and GANT61 in ccRCC cell lines (Caki-1, Caki-2, and SN12-PM6). (**g**) Wound healing assay depicted the effect of GLI1 inhibitors Erismodegib and GANT61 on the migration ability of caki-1. (**h**) Primary ccRCC tumor cells were stained with antibodies for Arl13b (red) to mark cilia, and DAPI (blue) to mark DNA. Scale bar = 10 μm.

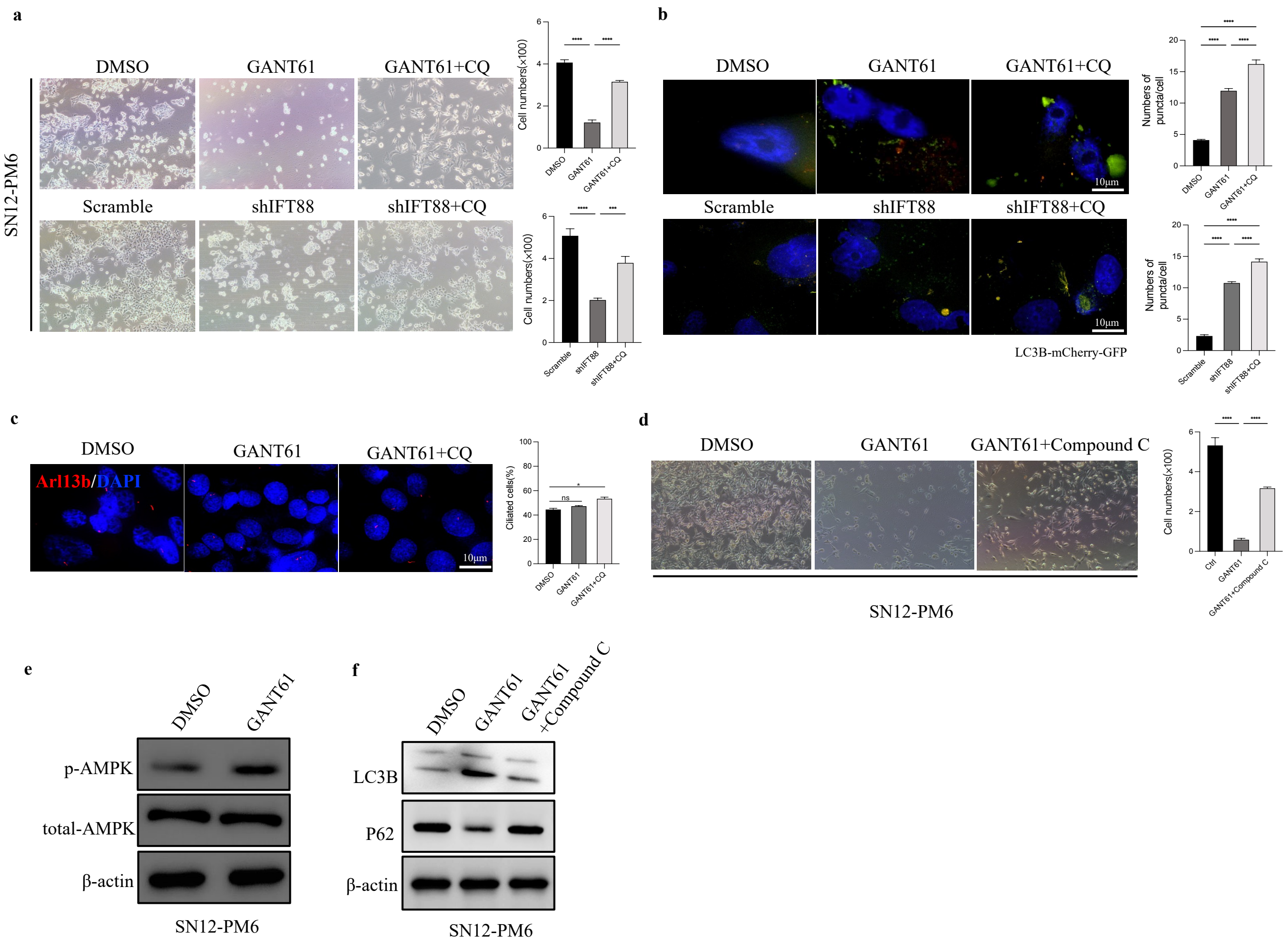

Figure S5 Chloroquine (CQ) and Compound C modulates autophagosome formation and cell growth.

(**a**) Representative bright-field images of SN12-PM6 cell transfected with shIFT88 or treated with GANT61 (5μM) with or without CQ (10μM). (**b**) SN12-PM6 cell was transfected with LC3B-mCherry-GFP followed by GANT61 treatment or IFT88 knockdown with or without CQ. LC3 puncta was visualized using confocal microscopy. Hoechst 33342 was used to stain the nuclei of cells (blue) . Scale bar = 10 μm. (**c**) Primary cilia expression using confocal microscopy in SN12-PM6 treated with DMSO, GANT61 and CQ. Cilia expression was determined using Arl13b-specific antibodies (red). Hoechst 33342 was used to stain the nuclei of cells (blue). Scale bar = 10 μm. (**d**) Representative bright-field images of SN12-PM6 cell treated with DMSO, GANT61 and AMPK inhibitor (Compound C) (10 μM). (**e**) Western blot assay of phospho-AMPK and AMPK in SN12-PM6 treated with DMSO or GANT61. (**f**) Western blot assay of LC3 and P62 in SN12-PM6 treated with DMSO, GANT61 and Compound C. *P < 0.05, **P < 0.01, ***P < 0.001, ****p < 0.0001.

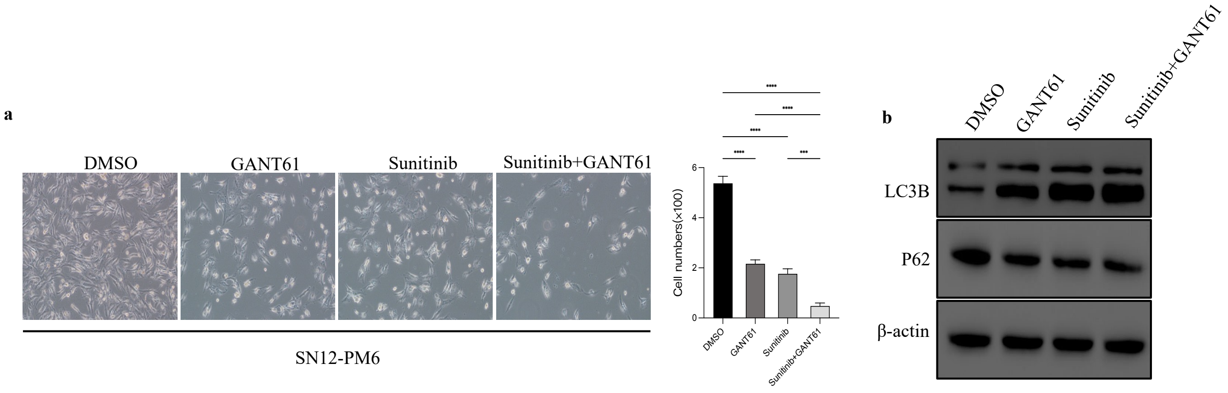

Figure S6 Sunitinib and GANT61 affects ccRCC cell growth and autophagy flux.

(**a**) Representative bright-field images of SN12-PM6 cell treated with DMSO, GANT61, Sunitinib, and their combination (10 μM). (**b**) Western blot analysis of LC3 and P62 in SN12-PM6 cells treated with DMSO, GANT61, Sunitinib, and their combination. *P < 0.05, **P < 0.01, ***P < 0.001, ****p < 0.0001.
