# Supplementary material for: Inhibition of primary cilia-hedgehog signaling axis triggers autophagic cell death and suppresses malignant progression of VHL wild-type ccRCC
Source: Cell Death Dis. 2024 Oct 10;15(10):739. doi: 10.1038/s41419-024-07085-8 (PMC11466958; doi:10.1038/s41419-024-07085-8)

Figure1

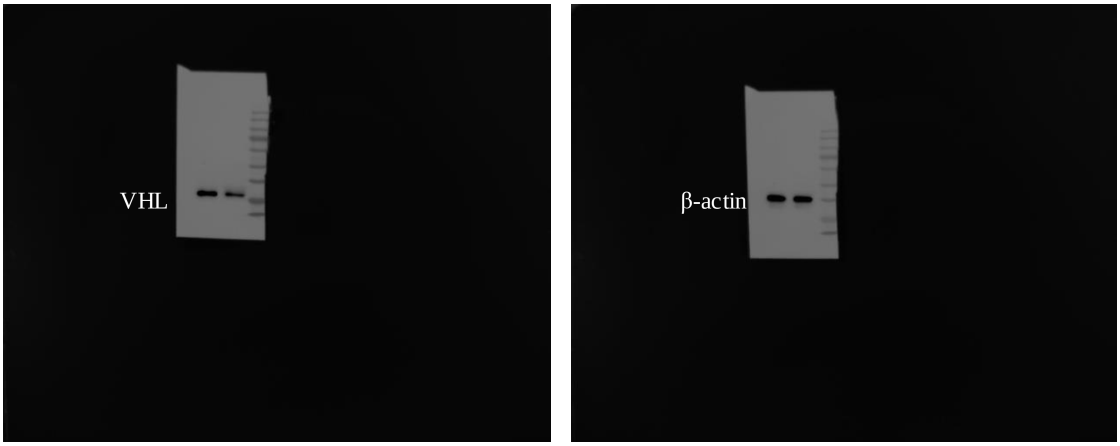


Figure2

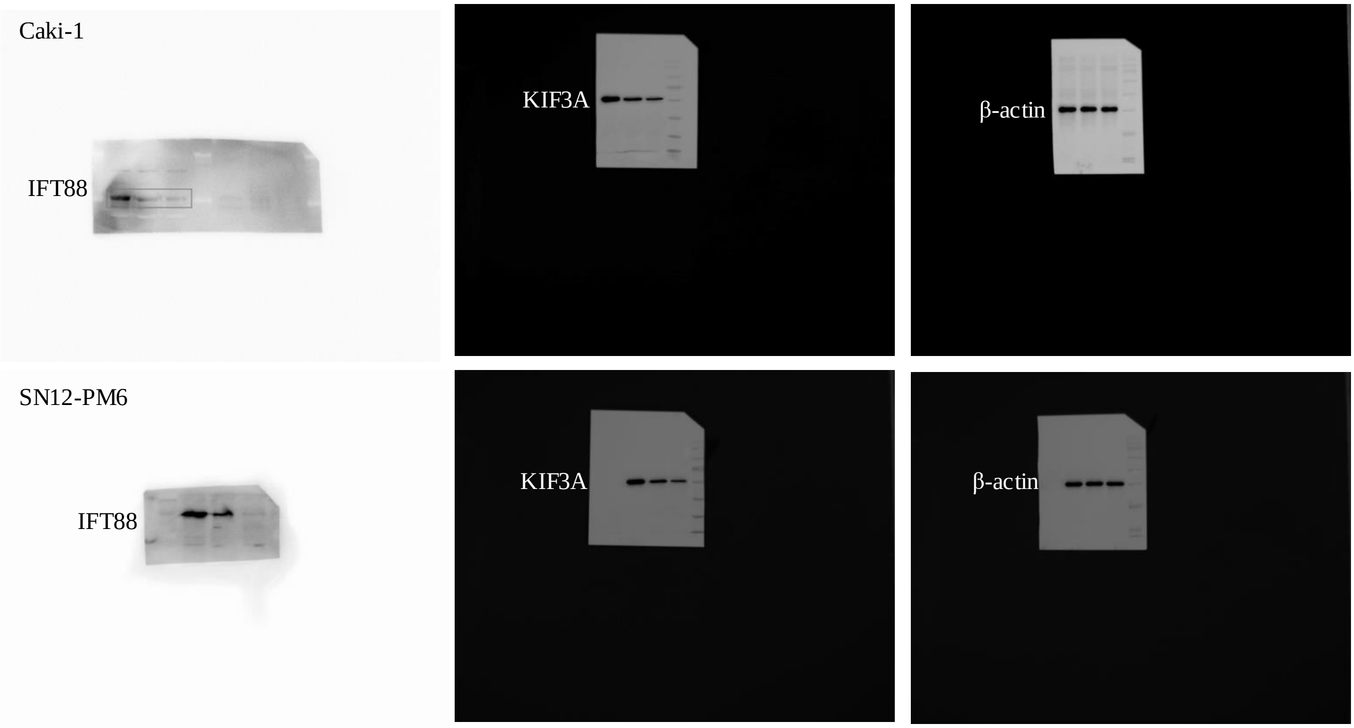


Figure3

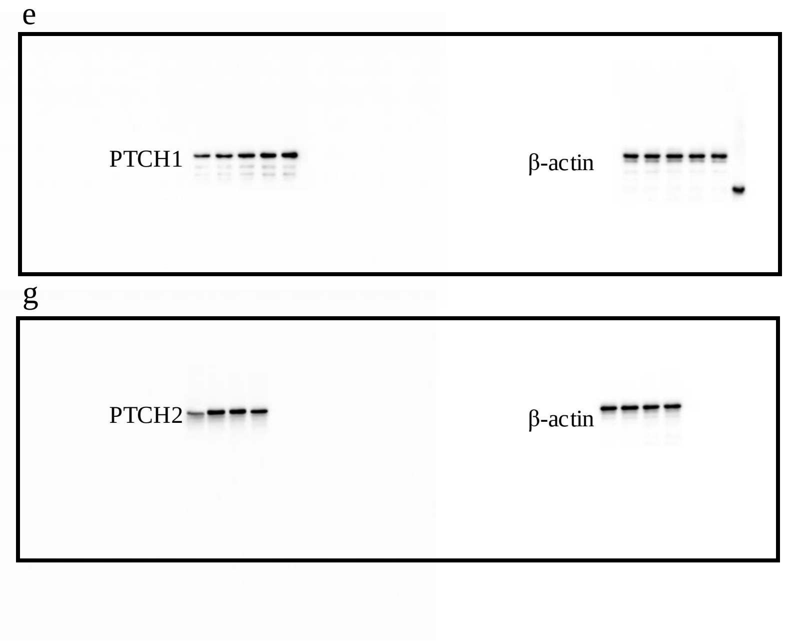

Figure6

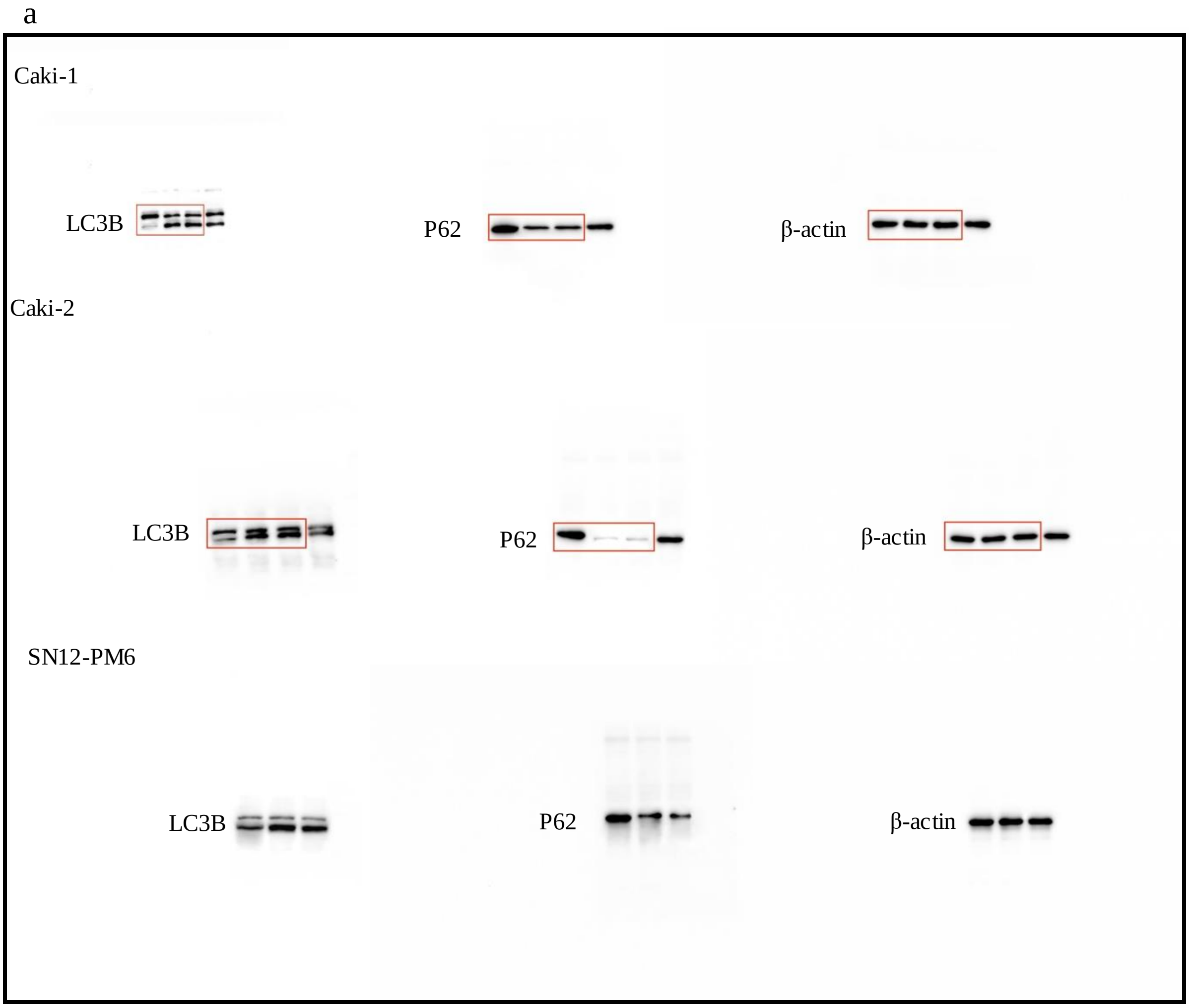
a
Caki-1
LC3B
P62
β-actin
Caki-2
LC3B
P62
β-actin
SN12-PM6
LC3B
P62
β-actin

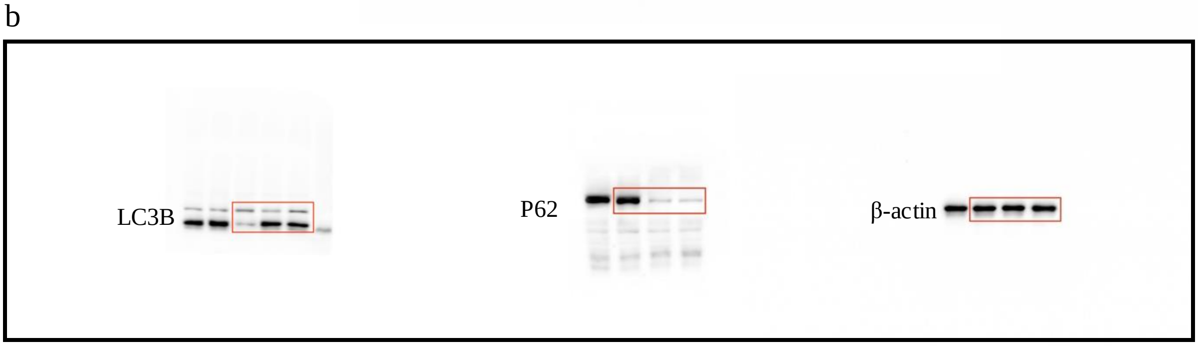
b
LC3B
P62
β-actin

Figure6

g

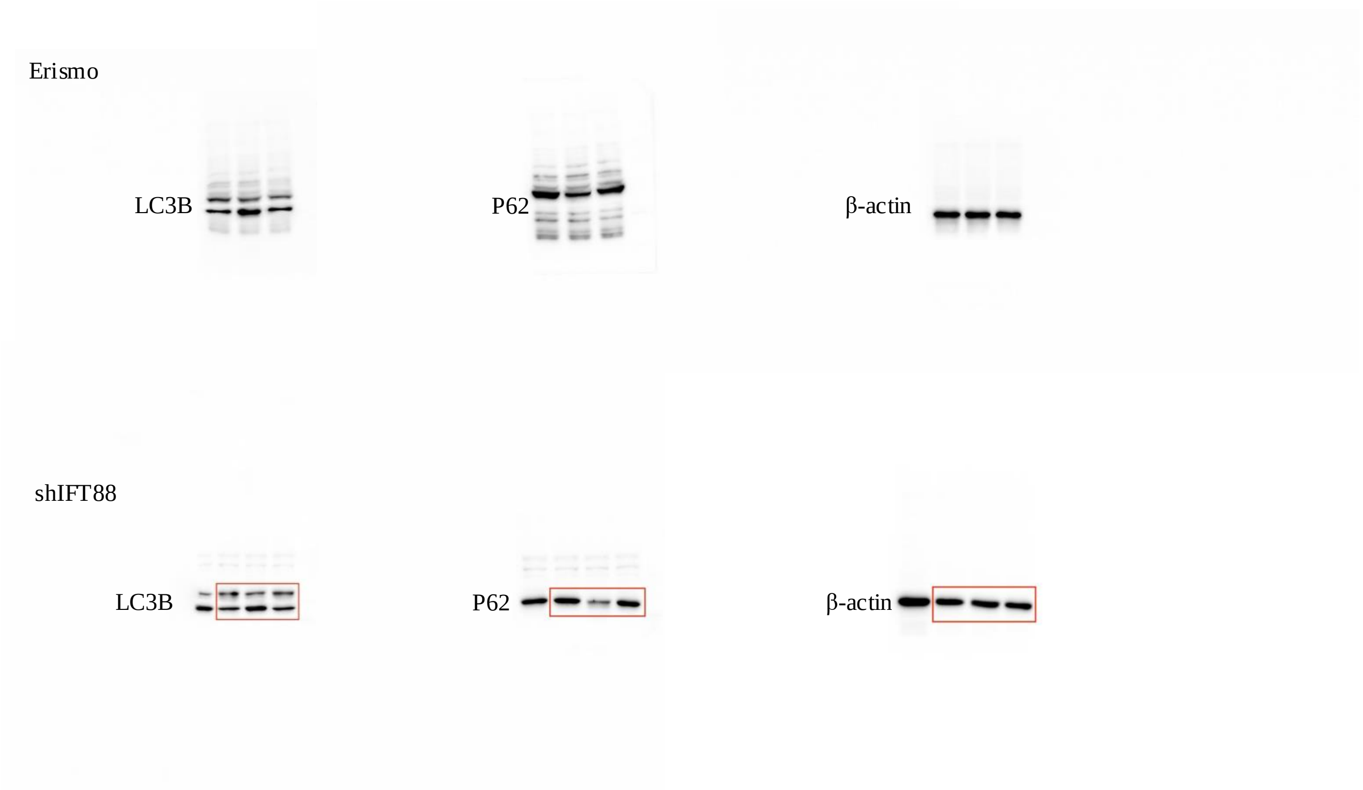


Supplementary figure4

c

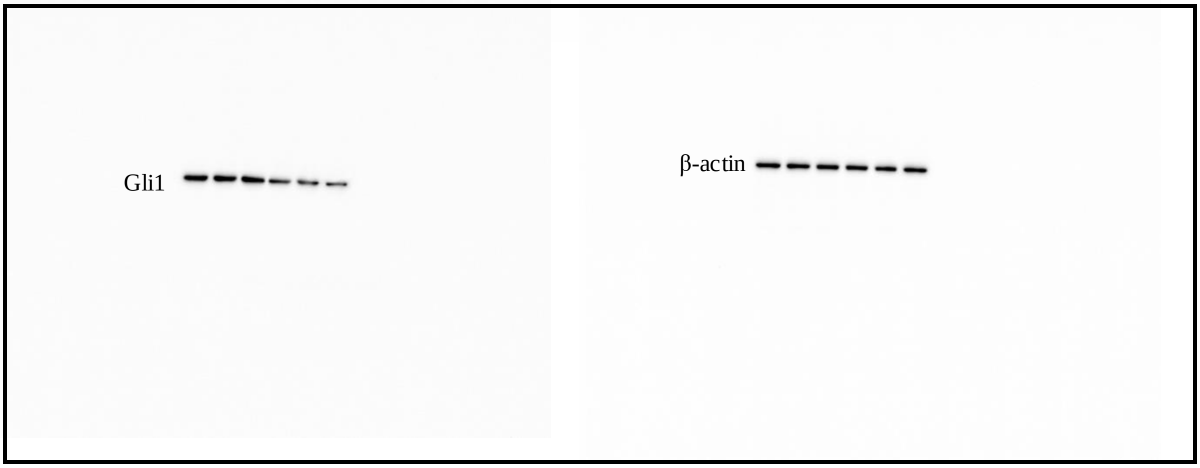


d

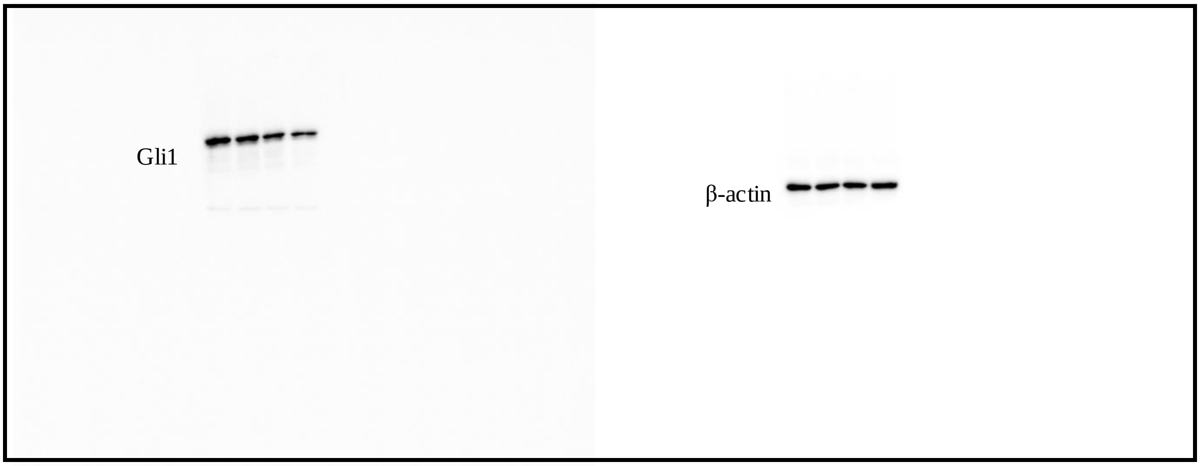

Supplementary figure5

e

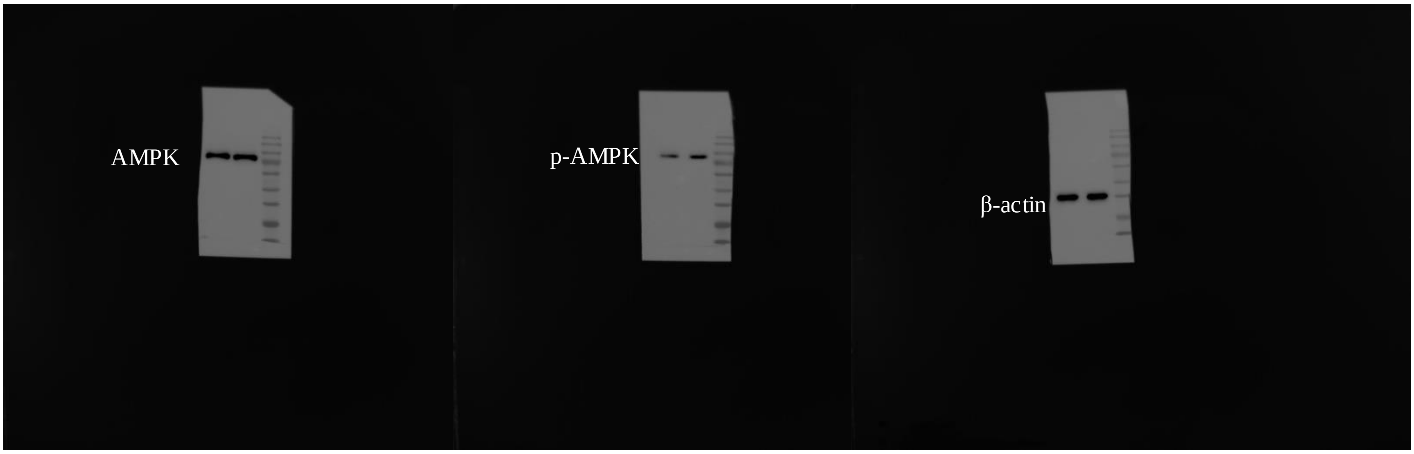


f

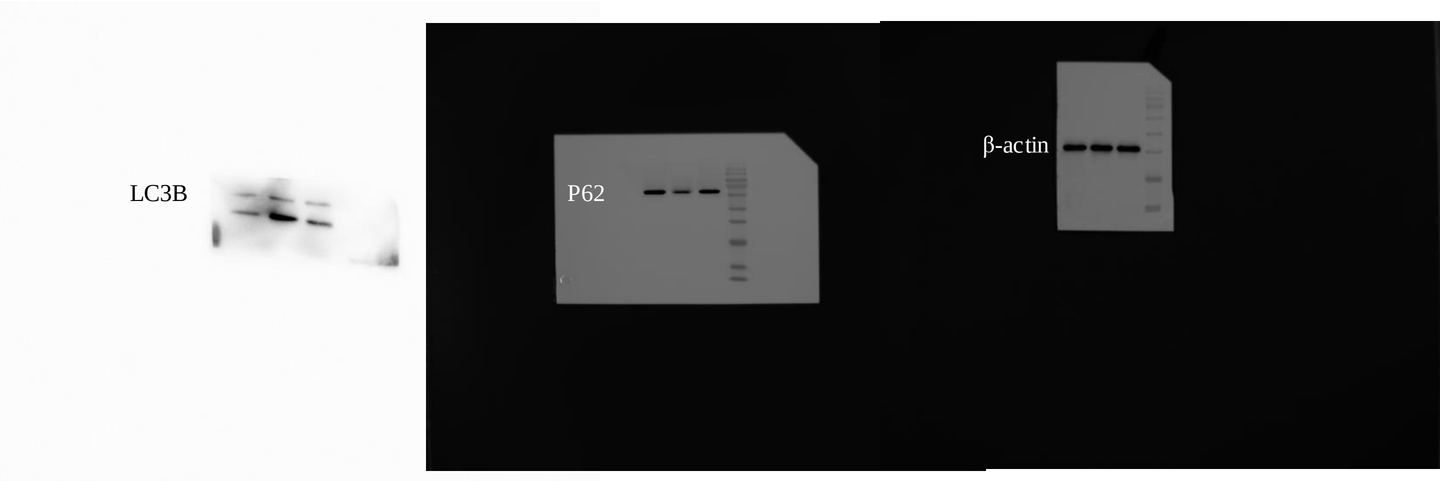


Supplementary figure6

b

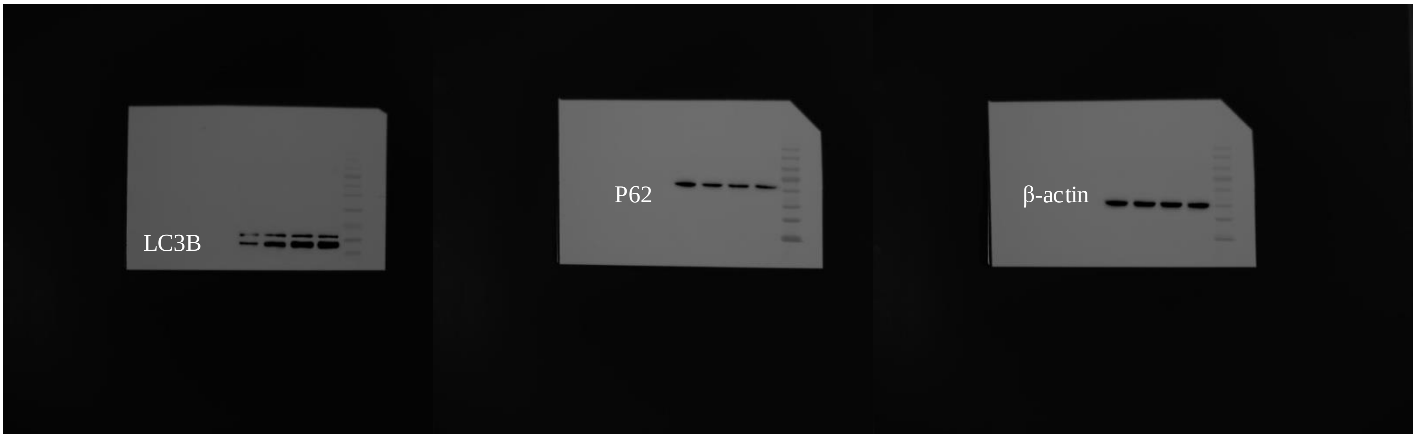

Supplement: Supplementary file 3 — Original data [file 41419_2024_7085_MOESM3_ESM.pdf]
